# Identifying magnetic phases in chemically ordered and disordered FeAl thin films†

**DOI:** 10.1039/d4ra06100d

**Published:** 2024-11-18

**Authors:** A. Zarzycki, M. S. Anwar, R. Bali, K. Potzger, M. Krupinski, M. Marszalek

**Affiliations:** a Institute of Nuclear Physics Polish Academy of Sciences Radzikowskiego 152 31-342 Krakow Poland; b Helmholtz-Zentrum Dresden-Rossendorf Bautzner Landstrasse 400 01328 Dresden Germany k.potzger@hzdr.de

## Abstract

Direct magnetic writing of ferromagnetic nanoscale elements provides an alternative pathway for potential application in data storage or spintronic devices. Magnetic patterning due to local chemical disordering of Fe_60_Al_40_ thin films results in adjacent nanoscale regions that possess two different phases, *viz.* a low-magnetization and high-coercive chemically ordered phase (non-irradiated ferromagnetic area, NIFM) and a high-magnetization and low-coercive chemically disordered phase (irradiated ferromagnetic area, IMF). Depending on the volume of NIFM and IFM phases, different interaction mechanisms were revealed. It was shown that the modulated films of the coexisting magnetic phases do not lead to exchange coupling in most cases. Evidence for exchange-spring behaviour, however, was found. Moreover, both magneto-structural phases at low temperatures show spin-glass-like properties. Understanding the influence of chemical ordering on magnetic properties is crucial for the advancement of the functionalities of spintronic devices and for the development of alloys with controllable magnetic properties.

## Abstract 2

Ferromagnetic (FM) and spin-glass (SG) phases coexist in spatially modulated chemically disordered Fe_60_Al_40_ thin films. Low-temperature magnetic interactions vary from uncoupled to exchange-spring behavior, depending on phase composition.

## Graphical abstract

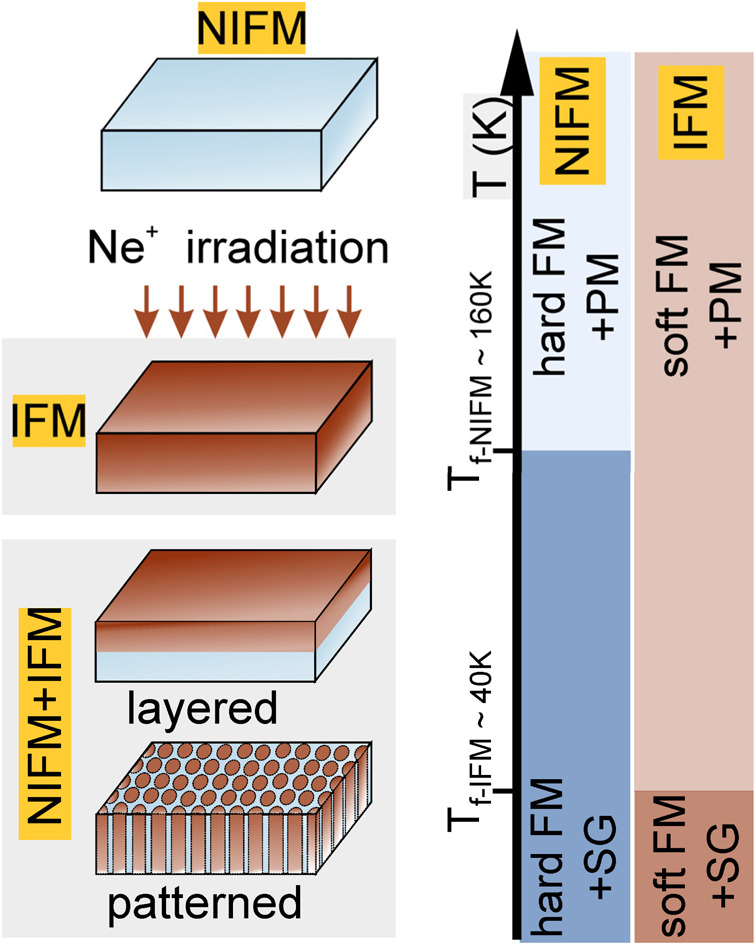

## Introduction

Patterning of ferromagnetic nanostructures from films is a powerful technique to fabricate size-scalable elements for magnetic data storage or spintronics. Usually, the nanostructures are lithographically produced as free-standing magnetic elements. Direct writing of ferromagnetic (FM) micro- and nanostructures into non-magnetic template films, however, bears several technological advantages, including a smooth surface. Such a process requires both an adequate template material for the lithographic process and a writing technique that alters the magnetic properties of the material but not its surface morphology. Low energy ion irradiation is a proper method to alter the structure of studied materials and meets the requirements for focusability, damage control and penetration depth. It can easily be applied to intermetallic alloys such as B2 Fe_60_Al_40_, short-range ordered Fe_60_V_40_ (ref. 1) and B2 Fe_50_Rh_50_,^2–4^ which are magnetically highly sensitive to a structural or chemical order, *i.e.*, they transform from non-ferromagnetic into ferromagnetic phases depending on structural or chemical defects. B2 chemically ordered Fe_60_Al_40_ alloy^5^ is of special interest because a very low ion fluence, *e.g.* of Ne^+^, is sufficient to force the Fe and Al atoms to swap their site occupancies. Thereby, the B2 ordered structure exhibiting negligible saturation magnetization (*M*_s_) at room temperature transfers into a chemically disordered, ferromagnetic A2 structure with an *M*_s_ of about 800 kA m^−1^. Mondal *et al.*^6^ highlighted that magnetic ordering originates from changes in the electronic structure between ordered and disordered phases. The process involves a change from itinerant to a more localized Fe 3d state in the hybridized Fe 3d–Al 3s and 3p valence bands. Thus far, Fe_60_Al_40_ irradiated thin films have been investigated with respect to their magneto-structural phase transition,^7^ open volume defects,^8,9^ magnetic domain formation,^10,11^ transport properties,^12^ magnetic nano-elements,^13,14^ paramagnetic–ferromagnetic interface,^15^ and magnetic patterning using shadow masks or focused ion beams.^16–18^ In this context, nanosphere lithography (NSL) is particularly interesting as a scalable and inexpensive approach for patterning and provides highly ordered structures over large areas. Recent investigations involving polystyrene nanospheres have been performed in the fields of plasmonics^19,20^ and medicine.^21,22^ In this study, NSL combined with Ne^+^ ion irradiation was applied for magnetic patterning of Fe_60_Al_40_ thin films, resulting in magnetically modulated layers.^18^

Spin-glass-like behavior (thermomagnetic irreversibility) has already been observed in both irradiated and non-irradiated Fe_60_Al_40_ alloy thin films.^12,18^ Non-equilibrium disordered Fe_100−*x*_Al_*x*_ alloys prepared by sputter deposition^23^ have been shown to exhibit ferromagnetic hysteresis loops at 4.2 K. The magnetic phase diagram reveals spin-glass-like behaviors between *x* = 30 and *x* = 70 for the films grown on water-cooled substrates and between *x* = 42 and *x* = 72 for those grown on liquid-nitrogen-cooled substrates, the latter films being suspected to be of a lower chemical order than that of the former. The maximum temperature for the spin-glass-like phase approximately amounts to 40 K. The spin-glass-like behavior below 50 K was reproduced in ^24^ using electrical resistivity measurements and alternating current (AC) magnetometry. Neutron diffraction on B2 ordered alloy crystals of Fe(34Al), Fe(40Al), and Fe(43Al) revealed incommensurate spin density waves.^25^ The authors of^26^ showed that B2 Fe_100−*x*_Al_*x*_ at *x* = 34 showing a thermomagnetic irreversibility in the ZFC-FC curves around 40 K does not exhibit either ferromagnetic or spin-glass state in the sense of exchange interacting frustrated atomic moments. Instead, they observed “small groups” of Fe atoms with magnetization directed opposite to the total magnetization at low temperatures. The orientation of these groups depends on the number of nearest neighbor Al atoms. While the discussion about the origin of the spin-glass-like behavior of Fe–Al alloys with both the chemically ordered B2 as well as the chemically disordered A2 structure is still controversial, the phenomenology appears to be similar across all publications.

In this paper, special attention is paid to the fact that magnetic patterning does not create free standing ferromagnetic nanostructures, but induces such structures in a surrounding of similar structural and chemical properties exhibiting certain magnetic properties as well. Thus, the influence of the surrounding material on the ferromagnetic nanostructures, and *vice versa*, must be investigated. The magnetic analysis presented here focuses mainly on low temperatures, where the chemically ordered samples show a weak ferromagnetic response and spin-glass-like behavior. In the following text, we abbreviate the non-irradiated state of Fe_60_Al_40_ as “NIFM” (non-irradiated ferromagnetic), the ion irradiation induced phase as “IFM” (irradiated ferromagnetic), and the magneto-structural transformation as “phase transformation”. We present our investigations on the magnetic properties of both the NIFM (B2) and IFM (A2) phases in Fe_60_Al_40_ in close contact. Large area vertical interfaces between these phases have been prepared by means of Ne^+^ ion irradiation, with and without polystyrene masks of different sizes.

## Experimental

The samples (Table 1) were fabricated on Si substrates thermally oxidized with 270 nm SiO_2_. Then 40 nm-thick Fe_60_Al_40_ films were deposited from an alloy target of the same composition by DC magnetron sputtering at room temperature. The sputter deposition was performed in an Ar^+^ atmosphere of 2.9 × 10^−3^ mbar at a deposition rate of 0.56 Å s^−1^. The base pressure of the chamber was 1.8 × 10^−9^ mbar. The Fe_60_Al_40_ sputter target purity was 99.9%. The deposition was followed by an annealing step in ultra-high vacuum (9 × 10^−10^ mbar) at 500 °C for 1 hour. Heating and cooling rates of the annealing chamber were 5 °C min^−1^ and 10 °C min^−1^, respectively. During the annealing process, the B2-phase of the Fe_60_Al_40_ films was formed [see ref. 16 for details]. Polystyrene nanospheres have been deposited using an aqueous suspension, a widely used method for material deposition.^27^ After nanosphere deposition, B2-phase Fe_60_Al_40_ films were irradiated using broad-beam Ne^+^ ions at the Ion Beam Center (IBC) of the Helmholtz-Zentrum Dresden-Rossendorf. Thin films irradiated at an energy of 25 keV and a fluence of 6 × 10^14^ ions per cm^2^ resulted in a disordered A2 phase over the whole film thickness with maximum saturation magnetization (sample FeAl_25 keV). The film irradiated at 10 keV at the same fluence was prepared for comparison (sample FeAl_10 keV). In this case, approximately half of the film volume was transformed into a ferromagnet. The penetration profiles of the Ne^+^ ions (not shown) were simulated using SRIM^28^ applying the model from ref. 16 relating the magnetization to the displacements per atom (dpa).

**Table 1:** Preparation parameters of the Fe_60_Al_40_ patterned films, including the etching time of the PS nanospheres, ion irradiation energy and fluence. FeAl_nonirr refers to the non-PS-patterned and non-irradiated films. FeAl_10 keV and FeAl_25 keV refer to the non-PS-patterned films irradiated with Ne^+^ ions at 10 keV and 25 keV, respectively. The last columns display saturation magnetization measured at 10 K and 300 K, respectively

Sample identifier	Etching (min)	Irradiation, Ne^+^, 6 × 10^14^ cm^−2^	Irradiated volume, SEM + SRIM (%)	Irradiated volume, MFM + SRIM (%)	*M*_s_@10 K (emu cm^−3^)	*M*_s_@300 K (emu cm^−3^)
FeAl_nonirr	No spheres	Non irradiated	0	0	107	80.7
FeAl_10 keV	No spheres	10 keV	63 (ref. 16)	63 (ref.^16^)	425.7	358.2
FeAl_25 keV	No spheres	25 keV	100	100	725.7	602.8
FeAl_0 min	0	25 keV	9	11.5	257.8	178.2
FeAl_3 min	3	25 keV	28	27.5	287	205.4
FeAl_6 min	6	25 keV	44	38.5	354	294.9
FeAl_9 min	9	25 keV	56	46.5	498.7	381.9

While the films FeAl_10 keV and FeAl_25 keV have been fully irradiated over the whole surface area, lateral modification of the magnetic properties was realized by depositing a monolayer of self-assembled polystyrene (PS) spheres on additional as-prepared films prior to irradiation. For this purpose, an aqueous suspension with monodisperse PS spheres of 762 nm in diameter (microparticles GmbH) has been applied to a Milli-Q deionized water surface, where a highly ordered hexagonal close-packed monolayer was created by self-assembly. Subsequently, the spheres were deposited on the Fe_60_Al_40_ film surface by slow water evaporation.^29,30^ Next, RF-plasma etching was applied, resulting in a controlled decrease in the particle size, but maintaining their original positions and arrangement on the sample surface. Plasma etching was performed in an atmosphere of oxygen (purity 99,9%) and argon (purity 99 999%) in a ratio of 2/1, at a pressure of ∼0.2 mbar and a temperature of approximately 30 °C with a chamber base pressure of 0.06 mbar.^31^ The assemblies of PS spheres served as a shadow mask during ion irradiation. In order to transform the Fe_60_Al_40_ patterned films along their entire thickness into the ferromagnetic phase (IFM), irradiation at 25 keV was applied, which guarantees full shadowing effect. The samples of different etching times were prepared, *i.e.*, of 0 minutes (min), 3 min, 6 min and 9 min, which enabled us to obtain PS sphere sizes between 762 nm and 530 nm. The etching time defines the size of the polystyrene spheres during Ne^+^ ion irradiation and thus the fraction of the IFM *vs.* the NIFM volume in the patterned film (Table 1). After irradiation, the PS spheres were removed from the samples by ultrasonic assisted lift-off in ethanol, leaving behind the magnetically modulated Fe_60_Al_40_ layer.

The magnetic properties have been characterized by a superconducting quantum interference device magnetometer MPMS XL SQUID (Quantum Design). The samples were analyzed by magnetization *vs.* magnetic field *M*(*H*) hysteresis loops measured at 300 K and 10 K. Additionally, recoil curves have been measured at 10 K. In those measurements, the sample was initially saturated in a positive field and then minor loops between negative magnetic field and 0 Oe were recorded. ZFC–FC curves were measured as follows: the samples were cooled from room temperature (RT) down to 10 K in zero applied magnetic field. Next, an external magnetic field between 20 and 400 Oe was applied and the magnetization (*M*) was recorded with the increase in temperature (*T*) while warming the system up to 350 K, providing the ZFC segment of the curve. For the FC measurement, the sample was cooled in the presence of the applied field and the *M*–*T* dependence was recorded. Alternating current (AC) magnetometry was performed from 10 to 350 K at a frequency of 10 Hz and an amplitude of 3 Oe at constant external magnetic fields between 0 and 400 Oe. All the measurements were performed for the magnetic field pointing along the sample plane.

Scanning electron microscopy was performed using Tescan VEGA 3. Atomic force microscopy (AFM) and magnetic force microscopy (MFM) were performed using a Bruker Dimension Icon (ScanAsyst) system. A tip with low-moment magnetic coating from Nanosensors was used for studying the patterned samples. The tip was magnetized before mounting on the scan head using a permanent magnet along the axis passing through the base and the apex. The imaging of 512 pixel × 512 pixel was done in tapping-mode AFM and lift-mode MFM (at a lift height of 30 nm).

## Results and discussion

**Fig. 1:**
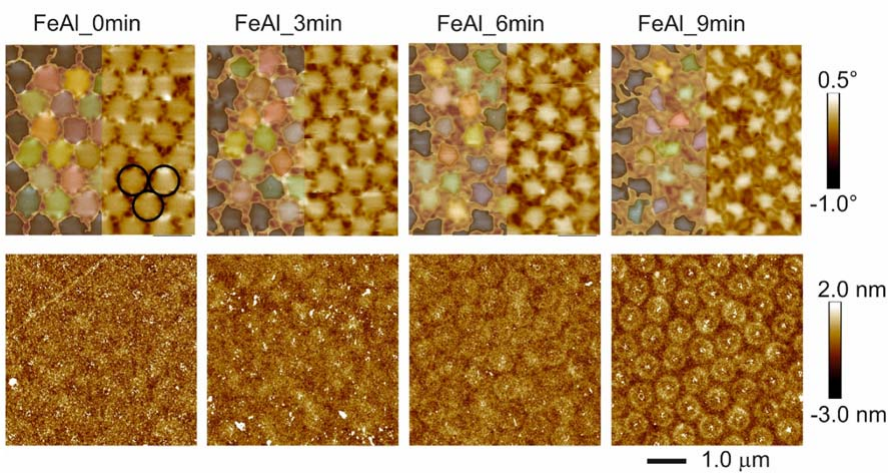
MFM (top row) and corresponding AFM images of the Fe_60_Al_40_ films irradiated through PS nanospheres. The original size of the spheres is indicated by black circles in the upper left figure. The identifiers of the samples indicate the etching time. Color scales for the phase (MFM) and height (AFM) are shown on the right. The MFM images (upper row) are overlaid with the shape/size of the non-irradiated areas (left half of each figure), estimated using.^32^

The effect of the irradiation through the PS nanosphere masks on the magnetic topology is shown in Fig. 1. The nanospheres self-assemble in a hexagonal pattern. After irradiation and removal of the spheres, the AFM exhibits flat surfaces except polystyrene traces of the deposited nanospheres, which become more pronounced with longer etching time. With the increase in etching time, the diameter of the nanospheres shrinks. After Ne^+^ ion irradiation, the IFM area between the nanospheres grows and becomes hexagonally arranged, NIFM vertical cylinders develop, surrounded by continuous ferromagnetic materials (IFMs) extending through the entire 40 nm-thick film. The ferromagnetic domain configuration is that of an in-plane soft-magnetic virgin state governed by the dipolar interaction between the ferromagnetic areas. For details on the domain configuration, please refer to ref. 18.

We calculated the irradiated volume by both a hard sphere model from the measured diameter of the spheres by SEM and by measuring the area appearing non-ferromagnetic in MFM using the ImageJ program.^32^ From Table 1 and Fig. S1,† it is evident that the irradiated area estimated from the diameter of the nanospheres is similar to the one obtained from MFM.

**Fig. 2:**
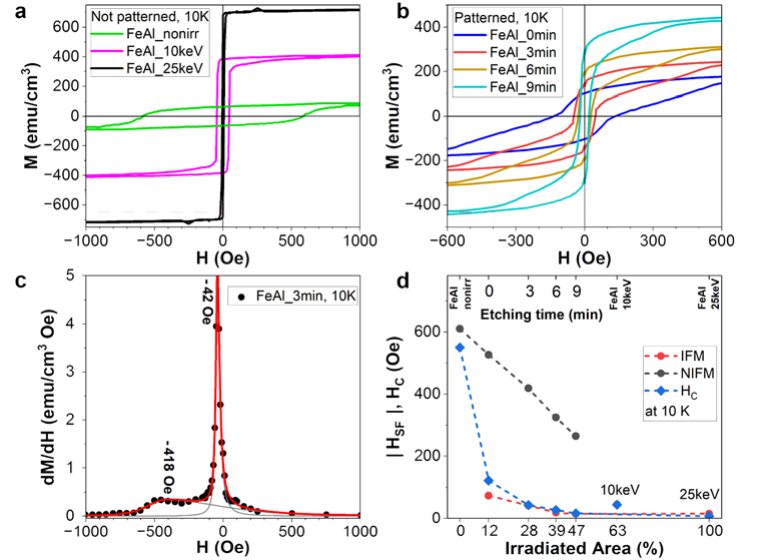
Integral magnetic properties measured using SQUID. All magnetization values refer to the total film thickness: (a) 10 K hysteresis loops for the non-patterned films, *i.e.*, the non-irradiated and the full area irradiated ones, (b) hysteresis loops for the patterned films, (c) exemplary depiction of the derivative d*M*/d*H vs.* magnetic field for sample FeAl_3 min, showing two switching fields. (d) Derived switching fields for all samples, along with *H*_C_. Usually, the onset of soft layer reversal at low negative fields is *H*_n_ (nucleation field), whereas the irreversible magnetization reversal field of the hard layer is the switching field (*H*_SF_). Here, we have referred only to switching fields *H*_SF_(NIFM) and *H*_SF_(IFM).

In order to characterize the integral magnetic properties of the samples, we performed magnetometry in the in-plane geometry at measurement temperatures of 10 K and 300 K. Starting with the non-irradiated sample FeAl_nonirr, with the increase in irradiated volume the hysteresis loops transfer from hard to soft magnetic, while the magnetization increases (see Fig. 2a, b and S2a and b†). We assign the development of the magnetization and the decrease in coercivity to the increase in the IFM fraction at the expense of the NIFM fraction in the films.

There is, however, a difference observable at 10 K between the fully irradiated films (Fig. 2a) and the patterned films (Fig. 2b). In the second and third quadrants of Fig. 2b, the patterned films show hysteresis curves, which consist of a steep decrease in the magnetization at low negative fields and a smooth decrease at higher fields related to irreversible magnetization rotation in the hard phase. In Fig. 2c, the dependence of the derivative d*M*/d*H* on *H* is shown exemplarily for FeAl_3 min, where the peaks represent distinct switching fields: *H*_SF_(NIFM) attributed to the non-irradiated ferromagnetic phase (larger negative value) and *H*_SF_(IFM) of the irradiated ferromagnetic phase (smaller negative value). Note that all the patterned films show two peaks, *i.e.*, two switching fields (Fig. 2d). Those samples consist of soft and hard magnetic phases. The asymmetry of the width of the d*M*/d*H* maxima shown in Fig. 2c hints towards a (non-rigid) coupling between the IFM and NIFM phases. Furthermore, the exchange coupling between these two phases leads to a decrease in *H*_SF_(IFM) and *H*_SF_(NIFM) with the fraction of the added IFM volume^33^ (Fig. 2d). The decrease in *H*_SF_(IFM), however, is small, while the decrease in *H*_SF_(NIFM) is more pronounced, *i.e.* adding the IFM volume softens magnetically the NIFM phase more than IFM. For the irradiated samples, *H*_C_ coincides with *H*_SF_(IFM), an effect similar to the one presented in Fig. 3d of ref. 34. The non-patterned, full-area-irradiated samples FeAl_10 keV and FeAl_25 keV, however, exhibit only soft fractions according to the switching field, *i.e.*, the constant behavior of *H*_SF_(IFM) for these films and the absence of *H*_SF_(NIFM) hint towards strong coupling or presence of soft layers only.

**Fig. 3:**
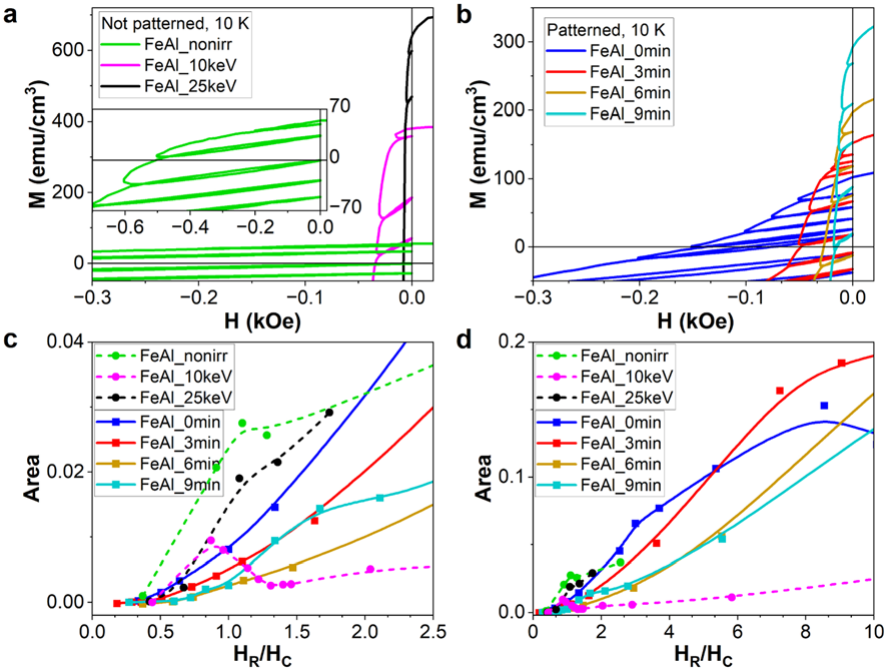
(a and b) Recoil loops for the non-patterned and patterned samples, respectively. The dependence of the hysteretic area within the recoil loops on the reverse field *H*_R_ relative to *H*_C_ and the preparation conditions are shown in (c and d). The inset in (a) shows the recoil loops for FeAl_nonirr samples in a larger range of magnetic fields.

Further investigating the magnetic coupling in the samples, we performed recoil loop measurements (Fig. 3a and b). For that, the samples have been saturated in-plane, applying a field of 50 kOe in a positive direction. After saturation, the field is lowered, until it reaches a certain negative value *H*_R_, called the reverse field. At this value of *H*_R_, the applied field *H* is increased again up to zero. In the next cycle, *H* is decreased again from zero until the next reverse field *H*_R_ is reached, where the recording of the recoil loop is repeated. The protocol ends well below the lowest reverse field value *H*_R_. Recoil loop curves have been recorded for several reverse field values crossing *H*_C_.

Recoil loop measurements are usually applied to layered or granular systems of adjacent soft and hard magnetic materials, giving rise to the formation of exchange-spring magnets. According to,^34^ irreversibility in the second quadrant of the FM hysteresis occurs for monophase materials. For single-phase magnetic systems, the recoil loops are not open. When the soft ferromagnetic phase is added to the hard phase, reversibility in the second quadrant is increased. Below a certain volume of the soft phase, they are rigidly exchanged, coupled and reversed simultaneously. For a sufficiently large volume of the soft phase, it switches reversibly at lower fields than the hard one, simultaneously reducing the hard phase's switching field. This state is called exchange-spring,^34^ where for magnetic fields above the negative switching field (of hard ferromagnet), the anisotropy is unidirectional. The amount of magnetization recovered upon removal of a particular reverse applied field characterizes the ‘‘springiness’’ of the exchange spring system.^35^ Increasing the ratio of the soft to hard phase further, they finally decouple and reverse independently. One of the main features under investigation is the openness of the recoil loops. In case of a layered system, the openness increases (as well as the recovery magnetization) with the increase in volume of the soft magnetic layer. At the same time, distinct switching and nucleation fields occur for the hard and soft layers, respectively (ref. 36 and ref. 5–10 therein). Further critical discussion on the role of exchange coupling, the presence of the soft magnetic phase and anisotropy variations on the recoil loop openness can be found in ref. 37–40.

In our case, different switching fields *H*_SF_(NIFM) and *H*_SF_(IFM) only occur at low temperatures, where the hard phase has a finite magnetization (Fig. 2c and d). In Fig. 3c, we show the evolution of the openness of the recoil loops measured by the hysteresis area with increasing *H*_R_ and dependent on the FM volume ratio between the IFM and NIFM phases for 10 K. The sample FeAl_nonirr is dominated by the hard phase. It shows only one switching field *H*_S_(NIFM) and a kink in the openness of the recoil loops at *H*_R_/*H*_C_ = 1 (Fig. 3c). Considering exchange interaction between soft and hard magnetic layers in exchange-spring systems, the openness of the recoil loop should increase with the increase in soft magnetic fraction, since the soft and hard volumes are increasingly more decoupled from each other. For our samples, the trend of the recoil hysteresis area does not appear to meet this rule. Instead, the openness decreases with the increase in IFM-to-NIFM volume ratio. Samples FeAl_nonirr, FeAl_10 keV, FeAl_25 keV, and FeAl_9 min show maxima or kinks of the recoil loop areas *vs. H*_R_ around *H*_C_ (Fig. 3c). Samples FeAl_0 min and FeAl_3 min exhibit kinks around the hard phase switching field *H*_SF_(NIFM), as visible in Fig. 3d. This indicates that, for the hard NIFM phase in the patterned samples with a short etching time, there is a residual openness of recoil loops. Once the film adopts the monophase IFM state, as observed for FeAl_25 keV, only one switching field is present in Fig. 2d. The openness remains below the value of FeAl_nonirr, but its dependence on *H*_R_ is similar. Thus, our system does not consist of volumes forming an ideal exchange spring magnet, but rather of a non-irradiated hard magnetic layer (NIFM) and a soft magnetic layer (IFM), both exhibiting poor reversibility and an open recoil loop. It should be noted that for all samples, the openness of the recoil loops increases with the increase in reversal field *H*_R_, which lies above *H*_C_, the latter being related to the hard phase (Fig. 3d). Sample FeAl_10 keV is an exceptional case since the area of the recoil loop reaches a maximum around *H*_R_ = *H*_C_ and then decreases with the increase in *H*_R_ (Fig. 3c). Additionally, there is no distinction possible between soft and hard phases (Fig. 2d). The film exhibits only one switching field; moreover, magnetization and coercivity are located between the values for FeAl_nonirr and FeAl_25 keV (Fig. 2a). Sample FeAl_10 keV consists of a NIFM bottom layer and an IFM top layer. Consequently, the architecture of FeAl_10 keV corresponds to a two-layered exchange-spring system. The presented recoil loops indicate that the investigated samples do not exhibit a typical exchange-spring behavior.

Moreover, the opened recoil loops are visible for FeAl_nonirr and FeAl_25 keV samples containing only NIFM and IFM phases, and hence, do not arise from exchange spring coupling between hard and soft ferromagnetic materials but are a result of other phenomena. The presence of open recoil loops was observed in inhomogeneous systems, for example, in nanocomposite materials.^41,42^ Therefore, we suspect that the chemical inhomogeneity of our samples can lead to thermomagnetic irreversibility, *i.e.*, spin-glass-like behavior. As discussed previously, the magnetic glassy behavior was repeatedly observed in FeAl alloys.^6,12,18,23–26^ The presence of a spin-glass phase characterized by a lack of long-range magnetic order would explain the continuous increase of recoil loop openness above the coercive field. To address this further, we performed thermomagnetic magnetometry as well as AC susceptibility measurements, which additionally allowed us to determine whether the inhomogeneity arises from superparamagnetic regions or magnetic frustration (atomic spin-glass).

**Fig. 4:**
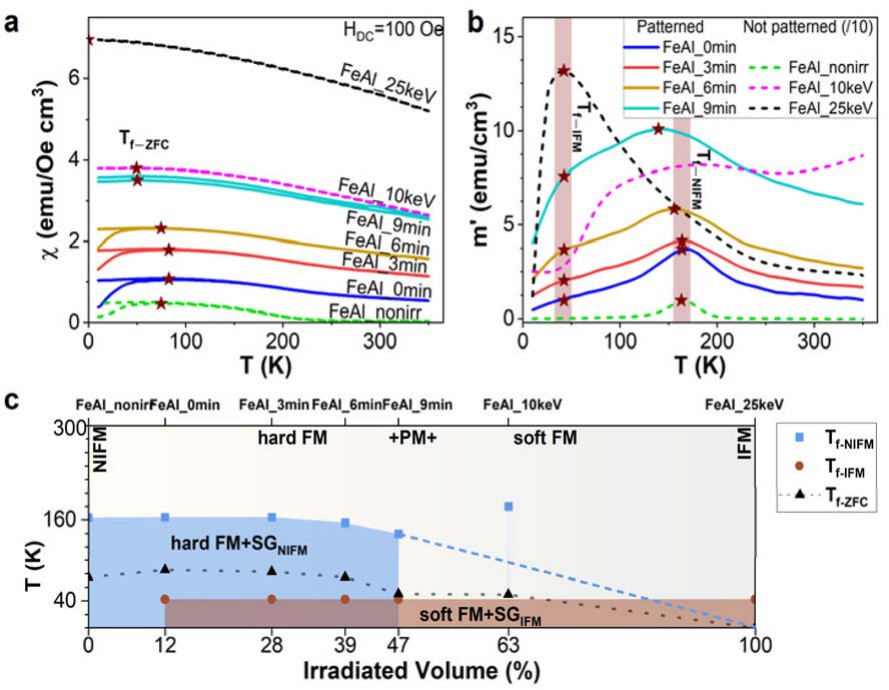
(a) ZFC–FC curves of magnetic susceptibility *χ* measured at an external field of *H*_DC_ = 100 Oe. The abbreviations of the samples are indicated above the curves. The maxima of the ZFC curves associated with the freezing temperature *T*_f_ are indicated by stars. (b) AC susceptibility at a zero external magnetic field and at a frequency of 10 Hz as a function of temperature. The stars show the evolution of *T*_f-NIFM_ and *T*_f-IFM_ with an increase in IFM volume. (c) Phase diagram of Fe_60_Al_40_ as a function of irradiated volume. The dashed blue line indicates the expected behavior of *T*_f-NIFM_.

An inhomogeneous, spin-glass-like magnetic system shows thermomagnetic irreversibility, characterized by several features: a splitting between ZFC and FC curves, a maximum of the magnetization in the ZFC curve, and the onset of hysteretic behavior during magnetization reversal, all occurring at specific temperatures. These three features typically manifest in the same temperature range but not necessarily at the exact same temperature. The freezing temperature *T*_f_, where the maximum in the ZFC curve is observed, is shown in Fig. 4a. It is notable that *T*_f_ decreases with the increase in irradiation volume, reflecting the phase transformation temperature from the NIFM phase to the IFM phase, the latter resembling a ferromagnetic system without thermomagnetic irreversibility. Fig. 4b displays the evolution of the real part *χ*′ of the AC susceptibility at zero external magnetic field and at a frequency of 10 Hz as a function of temperature. In our case, the freezing temperature *T*_f_ revealed from the ZFC curves splits into two components, which we can associate with the NIFM phase (*T*_f-NIFM_ ∼ 160 K) and the IFM phase (*T*_f-IFM_ ∼ 40 K). While *T*_f-NIFM_ decreases with the increase in the volume of the IFM phase, *T*_f-IFM_ remains constant. This is well seen in Fig. 4c, where the phase composition and its change with the irradiated volume are presented. The NIFM and IFM phases exhibit a weak ferromagnetic signal at room temperature and undergo the glassy ordering at low temperatures during cooling, meaning that at high temperatures, these phases contain ferromagnetic materials, together with paramagnet materials, while below the freezing temperatures, the paramagnetic part goes through the magnetic ordering. In the patterned samples, we can see a complex phase diagram with a mixed composition of NIFM and IFM phases. The figure also includes the *T*_f-ZFC_ dependence, which resembles the NIFM part but with lower values. Opposed behavior is found for the FeAl_10 keV sample (63% irradiated volume fraction). Here only one freezing temperature connected with the NIFM phase is present, which arises, as discussed previously, from exchange spring couplings in this double-layered system. The development of the temperature dependence of ZFC and *χ*′ with the increase in IFM volume is shown in Fig. S3 and S4† for different applied external fields. The relation between the AC susceptibility and magnetic relaxation time *τ* is typically well determined by the Debye model. Two main types of magnetic relaxation can be distinguished: the temperature-dependent magnetic relaxation, described by the phenomenological Vogel–Fulcher (VF) law, and the magnetization relaxation corresponding to an Arrhenius-type Néel relaxation for non-interacting superparamagnetic clusters. The distinction between both processes provides insights into whether the system is in a spin-glass or superparamagnetic state. This can be achieved by extracting the Mydosh parameter from *T*_f_ at different frequencies or by fitting the VF equation to the *T*_f_*vs.* frequency plot.^43–45^ For our samples, AC susceptibility measurements at frequencies other than 10 Hz were not possible because of the weak signal from the films.

**Fig. 5:**
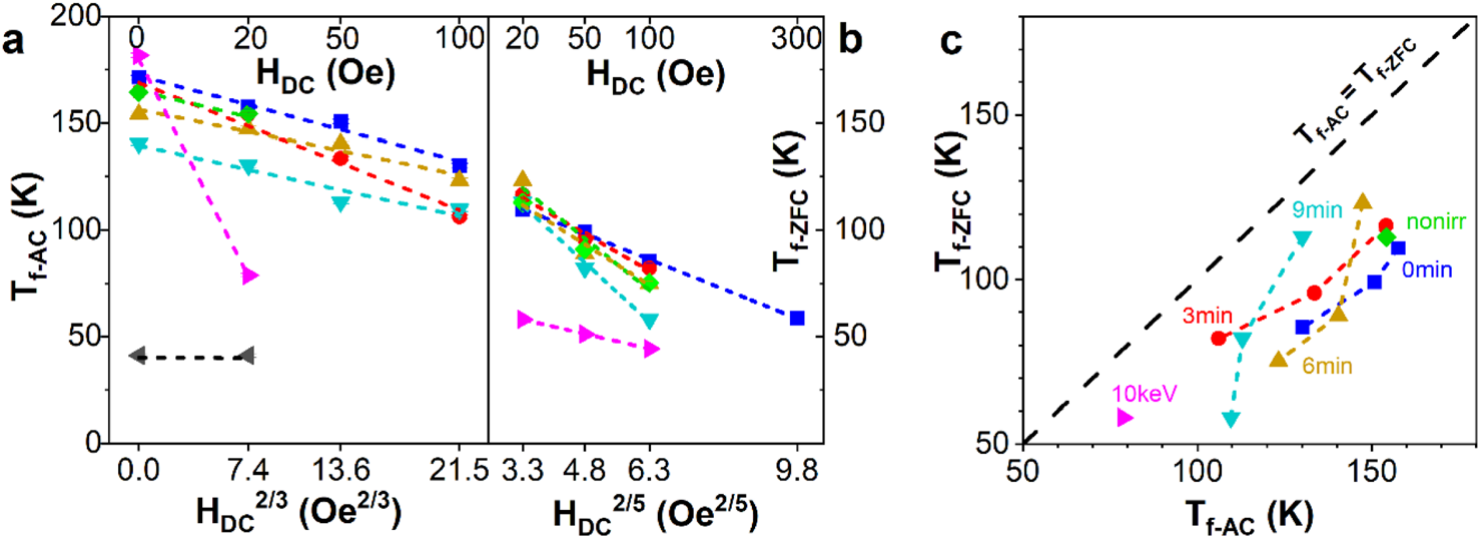
(a) *T*_f_*vs. H*_DC_^2/3^ derived from AC magnetometry and (b) *T*_f_*vs. H*_DC_^2/5^ of the ZFC/FC protocol. The development of *T*_f-ZFC_ is shown except for FeAl_25 keV, where the spin glass transition of the IFM phase is not observed. (c) Correlation of *T*_f_'s obtained from AC and ZFC curves.

Alternatively, the dependence of *T*_f_*vs. H*_DC_^2/*p*^ can be determined, as shown in Fig. 5a–c for both AC and ZFC/FC magnetometry measured. According to scaling theory with the mean field approximation,^46,47^ the value of the critical exponent *p* is expected to be 3 for the spin glass system, as found for *T*_f-AC_ in Fig. 5a. A similar analysis for *T*_f-ZFC_ shows an increase in *p* to 5, as shown in Fig. 5b. Such an increased exponent is found in the non-mean-field approach, see Table 1 in ref. 48. Therefore, we can conclude that regardless of the differences in scaling exponent *p* in the AC and ZFC studies, a spin-glass behavior occurs in both the NIFM and IFM phases. Based only on *T*_f_*vs. H* dependence, it is impossible to distinguish between different types of spin glass systems, but we can exclude the superparamagnetic effect being responsible for the bifurcation and presence of the maximum in ZFC/FC and AC measurements, since noninteracting systems such as superparamagnetic nanoparticles are expected to give a critical exponent *p* equal to 2.^49^

The discrepancy in *T*_f_(*H*) between AC and ZFC/FC measurements (Fig. 5a and b) is probably due to the complex phase composition of the samples, where both NIFM and IFM phases are composed of intermixed ferromagnetic and spin glass phases. Similar phenomena have been observed in other alloys such as iron-based Fe68V32 (ref. 50) and Fe_*x*_Cr_1−*x*_ (*x* = 0.2),^51^ furthermore a mixed state of spin glass and ferromagnetic phases has been theoretically shown to coexist, which explains phenomena found in alloys such as AuFe.^52^ In such material systems, the presence of ferromagnetic admixture is known to suppress the glassy transition while reducing the size of the cluster within the spin glass phase.^53^

To the best of our knowledge, there are no studies showing the effect of ferromagnetic admixture on the critical exponent *p* in the spin glass systems. With the increase in DC magnetic field, the influence from the ferromagnet becomes more dominant over thermomagnetic transition, although this effect varies between the two types of measurements. In a DC magnetic field, the spin glass transition is suppressed by the ferromagnet, with this being more pronounced in ZFC measurements compared to the AC ones. This effect is illustrated in Fig. 5c. The figure shows the correlation between the freezing temperatures obtained from AC and ZFC measurements and reveals a systematic shift of *T*_f-ZFC_ to lower values than those of *T*_f-AC_. This shift contributes to the difference in the critical exponent *p*, changing it from 3 for AC to 5 for ZFC studies, highlighting the fundamental difference between ZFC and AC magnetic measurements. The ZFC studies show agitation of magnetic moments with the increase in temperature in a constant DC magnetic field, whereas the AC studies show the response of magnetic moments to the AC magnetic field for the current temperature and DC magnetic field. Therefore, the ZFC curves provide insights into the magnetic response of the entire sample under thermal agitation at a specific magnetic DC field (including the spin glass transition and the influence of ferromagnetic part). The ferromagnetic part being magnetically coupled with the spin-glass phase intensifies the thermal agitations of the glassy phase, lowering the temperature of the transition. The influence of the ferromagnet is significant enough not only to shift the freezing temperature to lower values but also to suppress the second spin glass transition from the IFM phase, which is evident in AC studies. In contrast, AC susceptibility for such low frequencies predominantly detects the response from the spin glass phase and transition and is less sensitive to the ferromagnetic phase. This sensitivity difference appears because the AC field amplitude and frequency are tuned to the glassy transition response rather than the ferromagnetic phase.

## Conclusions

Polystyrene nanosphere lithography and irradiation with moderate ion fluences offer an alternative method to create magnetic patterning for data storage or spintronics. This approach allows for the fabrication of large area arrays of vertical, hexagonally arranged, low-magnetization ferromagnetic nanometer cylinders surrounded by continuous ferromagnetic materials. The diameter of these cylinders can be precisely controlled by the size of the polystyrene spheres used in the lithography process.

The study reveals the magnetic phases in chemically ordered and ion-irradiation disordered Fe_60_Al_40_ thin films. Moreover, it provides a comprehensive investigation on the interaction between adjacent phases depending upon their volume. The employed experimental techniques such as magnetometry enabled us to understand the mechanism of magnetic interaction.

We investigated the low-temperature magnetic behavior of Fe_60_Al_40_ thin films irradiated with Ne^+^ ions. The samples were prepared in the form of films, uniformly irradiated either with a broad ion beam or patterned samples, where ion irradiation took place through a monolayer of polystyrene microspheres. Despite the preparation method, all samples show strong inhomogeneity in magnetic properties.

The non-patterned, non-irradiated reference sample already contains hard ferromagnetic regions and undergoes a glassy transition at low temperatures. Upon full-area irradiation at 25 keV ion energy, the films become soft ferromagnetic. The magnetic architecture of the non-patterned sample irradiated with 10 keV Ne^+^ ions resembles a two-layered exchange-spring system. The patterned films exhibit two switching fields at low temperatures, indicating the presence of soft and hard magnetic phases. The exchange coupling between these phases results in a decrease in the switching fields with the increase in the fraction of the soft magnetic component fraction. Recoil loop measurements indicate that patterned samples do not behave as ideal exchange spring magnets, and exhibit poor reversibility. They also display magnetic characteristics typical for inhomogeneous glassy systems.

## Data availability

Data for this article titled “Identifying magnetic phases in chemically ordered and disordered FeAl thin films” by A. Zarzycki, M. S. Anwar, R. Bali, K. Potzger, M. Krupinski and M. Marszalek, including AFM/MFM and SQUID data, are available at RODARE at https://doi.org/10.14278/rodare.3110.

## Conflicts of interest

There are no conflicts to declare.

## Supplementary Material

RA-014-D4RA06100D-s001
