# Supplementary material for: Identifying magnetic phases in chemically ordered and disordered FeAl thin films
Source: RSC Adv. 2024 Nov 18;14(49):36763–70. doi: 10.1039/d4ra06100d (PMC11571056; doi:10.1039/d4ra06100d)
Supplement: RA-014-D4RA06100D-s001 [file RA-014-D4RA06100D-s001.pdf]

This journal is © The Royal Society of Chemistry 2024

Supplementary Material

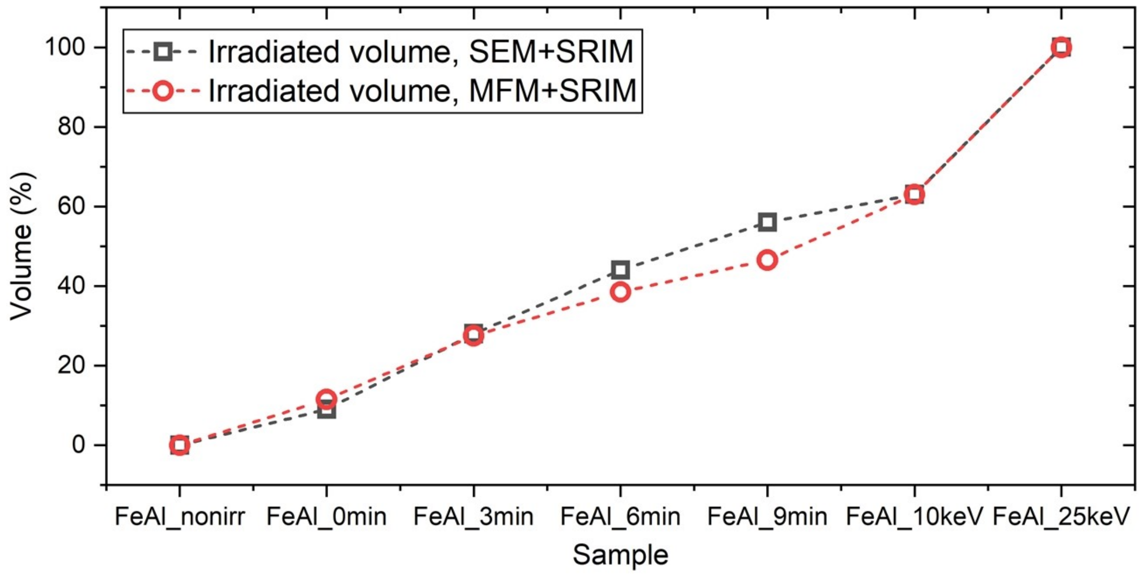

Fig. S1: Evolution of the irradiated and ferromagnetic volume fraction with etching time of the PS nanospheres. The two graphs compare the irradiated volume estimated from the SEM measurements of the spheres diameter and from the MFM measurements of the ferromagnetic area. The graph also contains the fraction for the $Fe_{60}Al_{40}$ thin film irradiated with 10 keV Ne^+ ions at 6×10^{14} cm^{-2}. The 25 keV Ne^+ irradiation was taken as 100 % ferromagnetic volume.

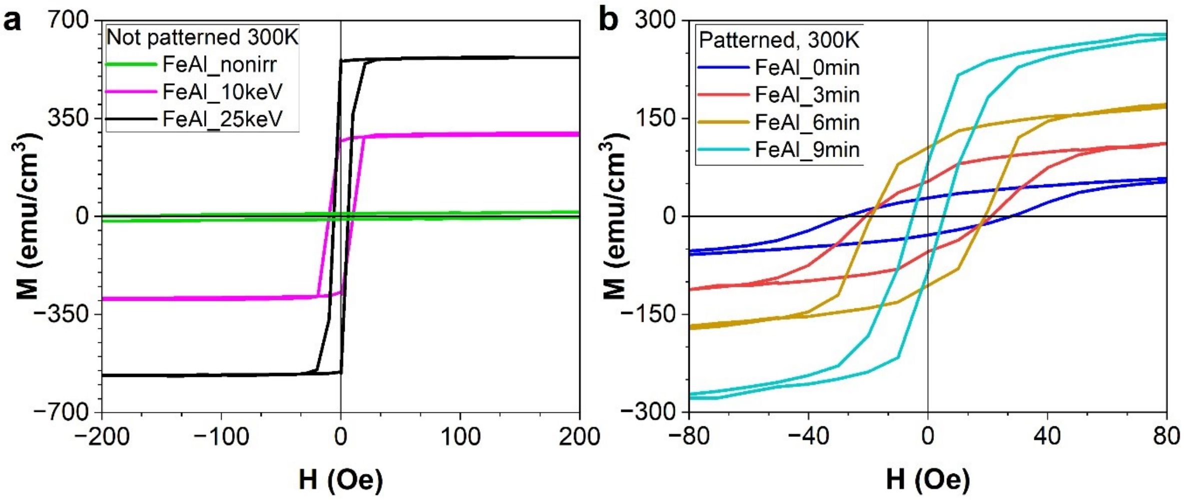

Fig S2. *The magnetization vs. field loops at room temperature for (a) the patterned films, (b) the non-patterned films, i.e., the non-irradiated and the full area irradiated ones.*

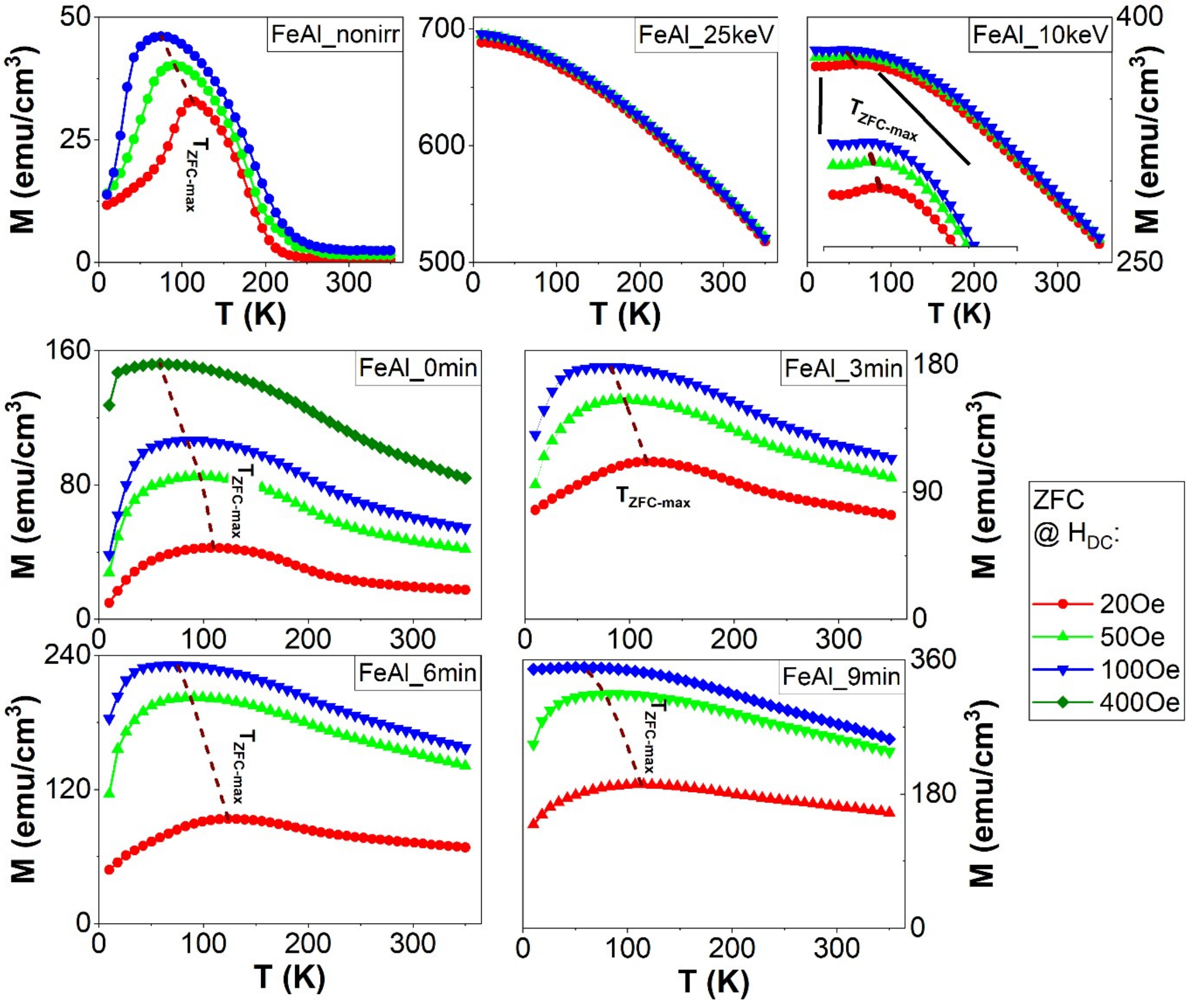

Fig. S3. *Development of the temperature dependence of ZFC for different H_{DC} fields with increasing IFM volume in the sample.*

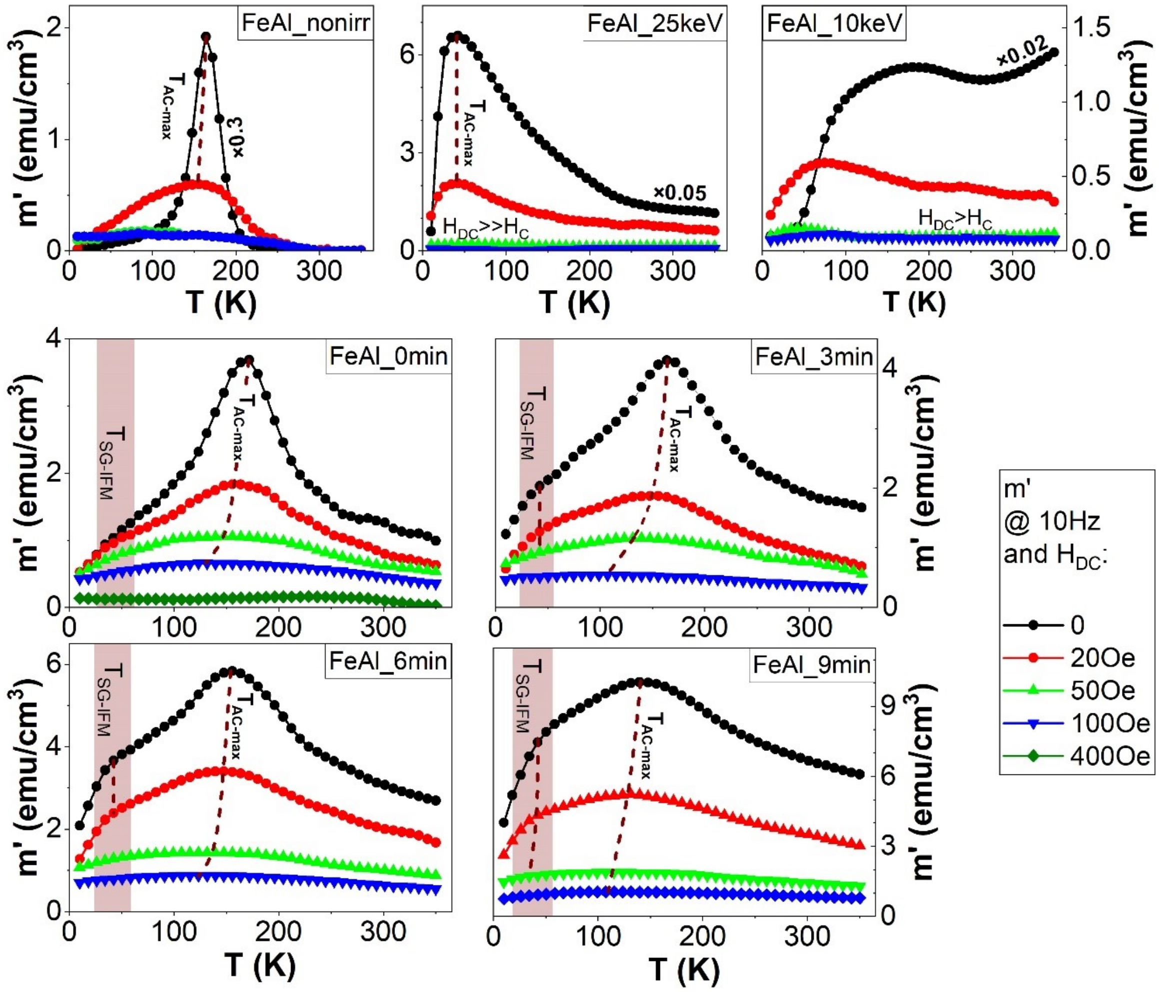

Fig. S4. *Development of the temperature dependence of χ' for different H_{DC} fields with increasing IFM volume in the sample.*
